# Quantifying beta cell function in the preclinical stages of type 1 diabetes

**DOI:** 10.1007/s00125-023-06011-5

**Published:** 2023-09-15

**Authors:** Alfonso Galderisi, Alice L. J. Carr, Mariangela Martino, Peter Taylor, Peter Senior, Colin Dayan

**Affiliations:** 1 Department of Pediatrics, Yale University, New Haven, CT USA; 2 Alberta Diabetes Institute, University of Alberta, Edmonton, AB Canada; 3 Division of Infection and Immunity, School of Medicine, Cardiff University, Cardiff, UK

**Keywords:** Diabetes stages, Metabolic modelling, Oral glucose tolerance test, Oral minimal model, Review, Stage 1 type 1 diabetes, Stage 2 type 1 diabetes, Type 1 diabetes

## Abstract

Clinically symptomatic type 1 diabetes (stage 3 type 1 diabetes) is preceded by a pre-symptomatic phase, characterised by progressive loss of functional beta cell mass after the onset of islet autoimmunity, with (stage 2) or without (stage 1) measurable changes in glucose profile during an OGTT. Identifying metabolic tests that can longitudinally track changes in beta cell function is of pivotal importance to track disease progression and measure the effect of disease-modifying interventions. In this review we describe the metabolic changes that occur in the early pre-symptomatic stages of type 1 diabetes with respect to both insulin secretion and insulin sensitivity, as well as the measurable outcomes that can be derived from the available tests. We also discuss the use of metabolic modelling to identify insulin secretion and sensitivity, and the measurable changes during dynamic tests such as the OGTT. Finally, we review the role of risk indices and minimally invasive measures such as those derived from the use of continuous glucose monitoring.

**Graphical Abstract:**

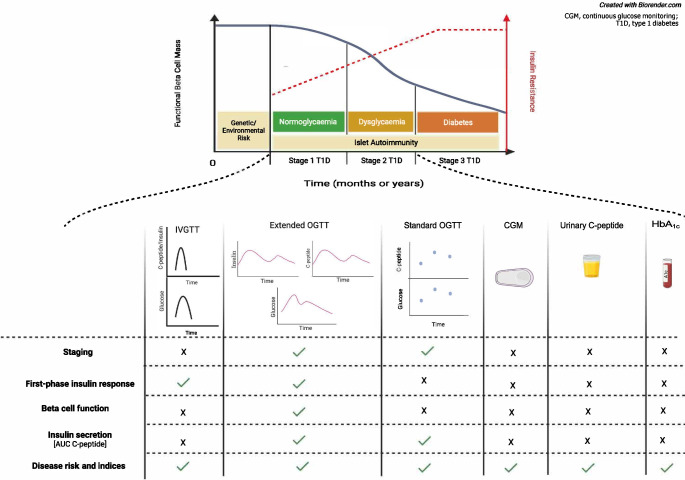

**Supplementary Information:**

The online version contains a slideset of the figures for download available at 10.1007/s00125-023-06011-5.

## Background

Symptomatic type 1 diabetes (stage 3 type 1 diabetes) is preceded by a prolonged pre-symptomatic phase characterised by progressive loss of functional beta cell mass after the onset of islet autoimmunity, with (stage 2) or without (stage 1) dysglycaemia during an OGTT [1]. Even in the absence of a measurable change in glucose profile during fasting or dynamic tests in early type 1 diabetes (stage 1), impairments of insulin secretion [2, 3] and insulin sensitivity [4–6] have been described, suggesting that beta cell impairment largely pre-dates increases in glucose and affects both insulin secretion and insulin action.

The approval of the first disease-modifying drug—the humanised anti-CD3 antibody teplizumab [7–9]—and extensive research on other agents [10] that may impact the trajectory of beta cell function highlight the need to identify effective measures of beta cell function. In this review we examine the metabolic changes that occur in the early stages of type 1 diabetes and the current methods for quantifying beta cell function, and discuss the possibility of longitudinally tracking the trajectory of beta cell function before progression to stage 3 type 1 diabetes.

## The importance of both insulin secretion and insulin sensitivity in evaluating beta cell health

**Fig. 1:**
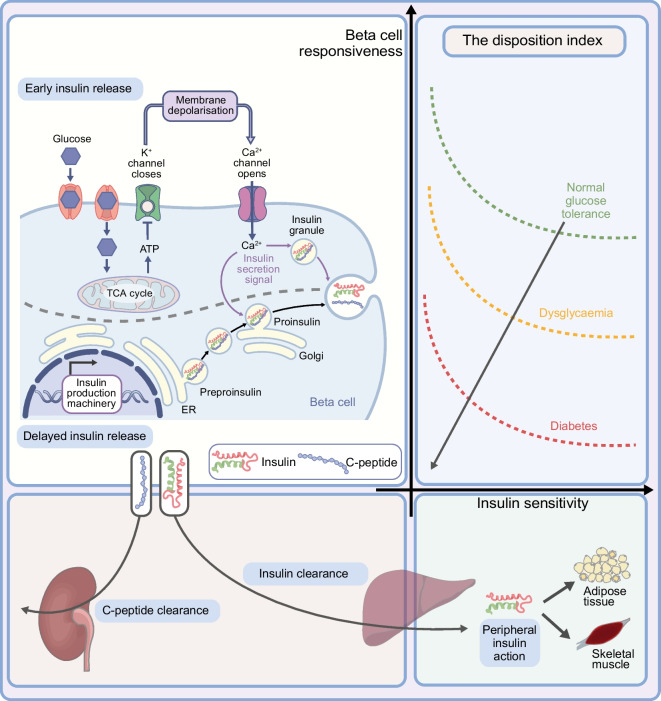
The DI results from the hyperbolic interaction of insulin secretion (beta cell responsiveness) and insulin sensitivity. Beta cell health can be quantified through the DI. Schematics representing insulin secretion and insulin sensitivity are shown. Early insulin release represents the response to a rapid glucose rise and is driven by the release of intracellular preformed insulin vesicles following glucose-induced beta cell depolarisation and calcium influx. This is followed by a second, delayed release of insulin in newly formed vesicles. Insulin and C-peptide are released in an equimolar ratio by the beta cell. Insulin is cleared by the first hepatic pass, with ~20% of the secreted insulin reaching peripheral target organs, while C-peptide is cleared by the kidney at a rate that is dependent on the GFR. The graph represents the dynamic interaction between insulin secretion and insulin sensitivity to maintain normoglycaemia. The transition from normal glucose tolerance to diabetes is shown by the failure to increase insulin secretion in response to changes in insulin sensitivity. ER, endoplasmic reticulum; TCA, tricarboxylic acid. This figure is available as part of a downloadable slideset

Beta cell function relies on two components, insulin secretion and insulin sensitivity, or, in other terms, on the ability of beta cell functional mass to deliver sufficient insulin to match whole-body requirements and to adequately respond to transient metabolic challenges to maintain normoglycaemia [11]. It is therefore essential to estimate both components to adequately describe beta cell health. The dynamic interaction between insulin secretion and insulin sensitivity is described by a hyperbolic curve that is quantified by the so-called disposition index (DI), namely the capacity of insulin to promote glucose disposal in target organs such as liver and muscle [12, 13]. The healthy beta cell has a substantial insulin secretory reserve that can dynamically match increases in insulin resistance throughout life, thus minimising glucose fluctuations. However, even in the absence of measurable impairment of the glucose profile, changes in insulin secretion and sensitivity have been described in the early stages of type 1 diabetes (Fig. 1) [2, 3, 14–16].

## Beta cell function in the early stages of type 1 diabetes : quantifying insulin secretion

### First-phase insulin response

**Fig. 2:**
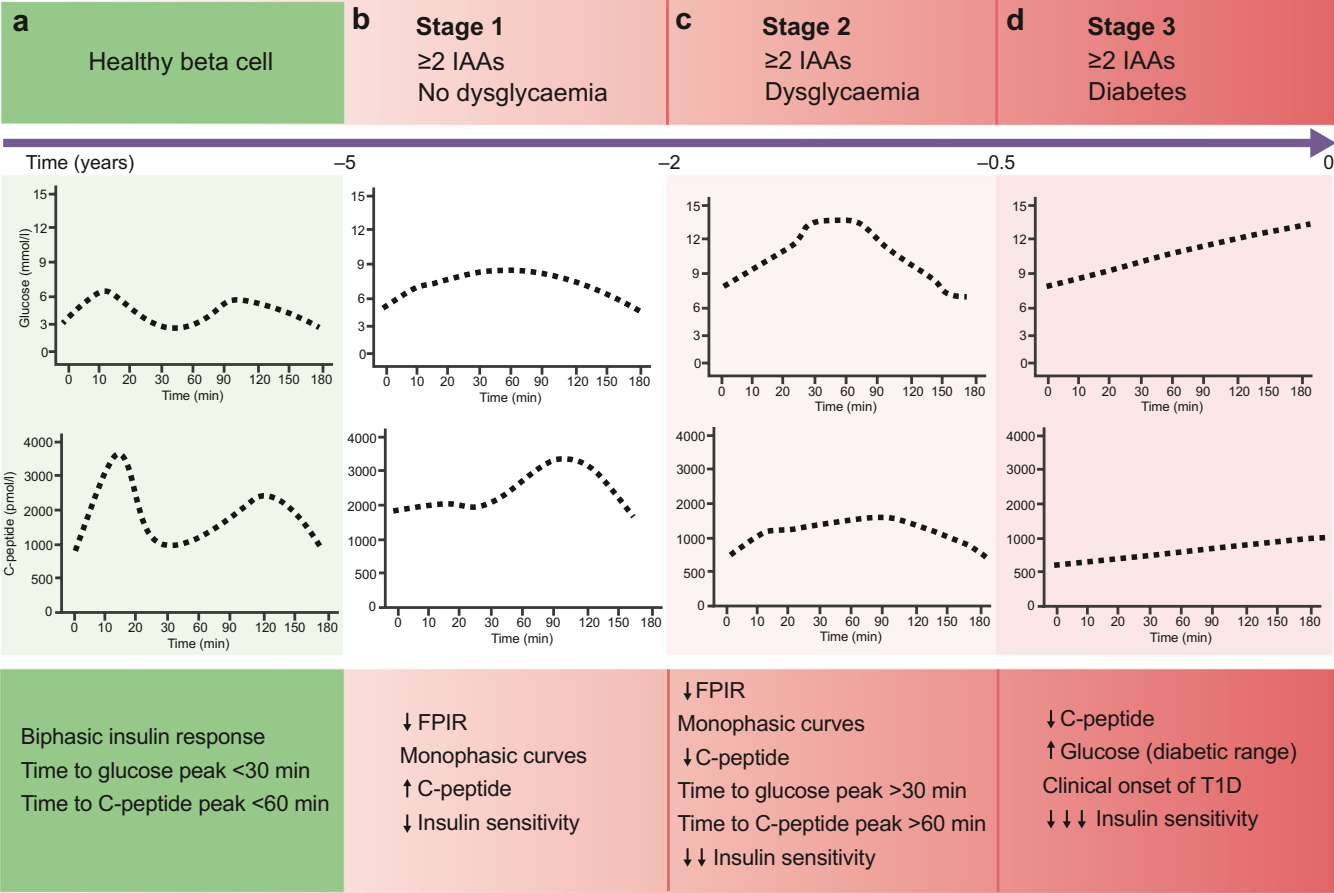
Glucose and C-peptide profiles during an OGTT through the progression from healthy beta cells (**a**) to stage 1 (**b**), stage 2 (**c**) and stage 3 (**d**) type 1 diabetes (T1D). The time (years) to diagnosis of type 1 diabetes is based on the available evidence and is intended as approximative. IAA, islet autoantibody. This figure is available as part of a downloadable slideset

The first-phase insulin response (FPIR) represents the response to a rapid glucose rise and is driven by the release of intracellular preformed insulin vesicles following glucose-induced beta cell depolarisation and calcium influx. The FPIR is classically assessed using an IVGTT, although a frequently sampled OGTT, with early samples taken during the first 30 min after the oral load, may also provide an accurate estimate [17, 18] (Fig. 2a). The loss of the glucose-induced FPIR is the most sensitive and earliest marker of beta cell dysfunction in type 1 diabetes [19–21]. While FPIR loss seems to be a marker of functional beta cell mass, some investigators have suggested that it may be secondary to insulin resistance and mild hyperglycaemia, therefore indicating that insulin resistance—and not secretion—may also be a primary driver for type 1 diabetes; however, this remains controversial [19].

The loss of the FPIR occurs 4 to 6 years before clinically symptomatic type 1 diabetes, accompanying islet autoantibody seroconversion [2, 22], and its measurement has been adopted to stratify diabetes risk in early prevention trials such as the Diabetes Prevention Trial – Type 1 Diabetes (DPT-1) [14] and the European Nicotinamide Diabetes Intervention Trial (ENDIT) study [23].

### Late compensatory changes in insulin secretion

The FPIR is physiologically followed by a second, delayed release of insulin in newly formed vesicles, which is represented by the biphasic insulin profile observed during an OGTT in an individual without diabetes [24, 25]. A compensatory increase in C-peptide secretion during this late phase of the OGTT (after 60 min) has been described following the initial loss of the FPIR [3, 26, 27] (Fig. 2b).

Data from the TrialNet Pathway to Prevention (TNPTP) study in relatives with at least two islet autoantibodies of people with type 1 diabetes describe a biphasic trend in AUC C-peptide during an OGTT before the clinical onset of type 1 diabetes, with a prolonged period of apparent stability of C-peptide or, in some studies, a ‘paradoxical’ increase in C-peptide levels [28]. This reaches a critical point ~6 months before the diagnosis of stage 3 type 1 diabetes, when the pattern changes to a rapid decline in C-peptide levels (Fig. 2b–d) [27, 28]. The compensatory increase in the late phase of insulin secretion probably accounts for the early stability or increase in overall AUC C-peptide release, masking a progressive decline in functional beta cell mass (Fig. 2b) [28]. While during stage 1 type 1 diabetes (Fig. 2b) the glucose excursion after an oral load resembles the healthy response, shown by biphasic glucose and C-peptide excursions, during stage 2 the compensatory delayed secretory response results in delayed glucose and C-peptide peaks, with monophasic glucose and C-peptide excursions (Fig. 2c). This is supported by the observation that a time to peak glucose >30 min [29] is associated with a higher risk of metabolic progression. A monophasic glucose curve (with one peak) was also more frequent in progressors to type 1 diabetes than non-progressors among antibody-positive individuals with serial OGTTs in the TNPTP study [30] (Fig. 2c).

## Beta cell function in the early stages of type 1 diabetes : quantifying insulin resistance

The role of insulin resistance in type 1 diabetes onset has long been debated. Suggestions range from it being a hypothetical disease accelerator [31] to being the primary determinant of beta cell failure [5, 19]. The exact temporal sequence of insulin secretion and insulin resistance changes remains uncertain. Seroconversion to multiple islet autoantibodies [32] parallels the transient physiological reduced insulin sensitivity described in healthy toddlers and adolescents, which peaks twice: before the age of 2 years, during the so-called mini-puberty, well described in toddlers and featuring hormonal changes comparable to those seen in the actual pubertal transition [33], and after the age of 10 years, during the pubertal transition [13].

Early observations conducted in a subgroup of asymptomatic participants in the DPT-1 trial, exhibiting fasting hyperglycaemia and 2 h glucose values >11.1 mmol/l, demonstrated reduced insulin sensitivity [34]. Similarly, a quantitative assessment of insulin resistance in the DPT-1 cohort based on the model of Mari et al [35] also demonstrated lower insulin sensitivity in those who progressed to clinical diabetes, in the absence of a difference at baseline [5], with a steep decline in insulin sensitivity 1 year before the diagnosis of clinically symptomatic type 1 diabetes [6]. More recently, a lower insulin sensitivity has been described in those with islet autoimmunity in the absence of dysglycaemia (stage 1) compared with their healthy peers [4]. Insulin resistance has also been described as a feature of type 1 diabetes in the absence of traditional risk factors, with the use of a euglycaemic clamp in lean individuals achieving glucose control targets [36–38].

## Measuring beta cell health: static vs dynamic tests

Traditionally, measurement of insulin secretion relies on C-peptide plasma concentrations during static (at a single time point) or dynamic (over multiple time points) tests. Up to 80% of insulin secreted in the portal vein is cleared by the liver, with large variability depending on the population studied [39, 40]. On the other hand, C-peptide, secreted in an equimolar ratio with insulin, is not subject to hepatic first-pass clearance and exhibits a longer half-life than insulin (~30 min vs ~4 min) [41], with an almost constant peripheral clearance [41–43]. These characteristics make C-peptide, rather than insulin, the almost ideal analyte for estimating insulin secretion.

C-peptide can be measured in a fasting or non-fasting (random) sample as a simple clinically deployed static test [44]. Dynamic testing, on the other hand, refers to tests such as the OGTT and mixed meal tolerance test (MMTT), which require a glucose challenge (and amino acid challenge in the case of the MMTT) and longitudinal measurement over 2–4 h.

### Static tests

Static measures of fasting glucose and C-peptide provide some evidence for actual disease progression in those in the early stages of type 1 diabetes [4, 45]. An early increase in postprandial glucose detectable up to 2 months before seroconversion in paediatric at-risk cohorts has been described [46]. In addition, a longitudinal analysis of The Environmental Determinants of Diabetes in the Young (TEDDY) study and the TNPTP cohorts, including children with islet autoimmunity, demonstrated that a 10% rise in HbA_1c_ in the non-diabetic range was as accurate as 2 h glucose values after an OGTT as a predictor of disease progression to stage 3 type 1 diabetes [47, 48].

The serum proinsulin-to-C-peptide ratio has been demonstrated to be predictive of type 1 diabetes, with higher fasted ratio in progressors ~1 year before clinical onset of type 1 diabetes in the TNPTP cohort [49] and others [50] as a consequence of derailed insulin processing but appeared to change little over time [8]. The urinary C-peptide-to-creatinine ratio may also serve as a surrogate marker of beta cell function [51, 52], with the possibility of capturing insulin resistance, as demonstrated in healthy individuals [53, 54], but has not been studied longitudinally in early-stage type 1 diabetes.

Measurement of random serum C-peptide levels in people with new-onset type 1 diabetes [55, 56] and established type 1 diabetes [57] have confirmed clear differences in disease progression between children and adults. Even among children, those diagnosed with type 1 diabetes at the youngest ages (<7 years) are frequently seen to progress to absolute insulin deficiency more rapidly [55], with many having undetectable levels of C-peptide at diagnosis, which has been shown to mirror the quantifiable beta cell mass in histological studies [55, 57]. As sampling and detection methods have evolved, single measurement of C-peptide levels in blood has become a cheap and easily accessible test, with a recent study demonstrating how transdermal capillary blood collection for C-peptide measurement is a reliable alternative to venous sampling [58].

However, limits of static tests for quantifying the subtle changes in beta cell function in early-stage type 1 diabetes remain. Although these tests are convenient and have some value after diagnosis, they are generally not sufficiently informative to be used alone either in cohort studies or in interventional trials prior to diagnosis.

### Dynamic tests

The OGTT remains the gold standard for staging type 1 diabetes according to the current classifications [1, 48, 59] and is the favourite of most screening programmes [30, 59–61], while the MMTT has been mostly used to monitor beta cell function after clinical onset of the disease (early-stage 3 type 1 diabetes) [62, 63]. From a physiology standpoint, while the OGTT measures insulin secretion in response to a single secretagogue, oral glucose, the MMTT also includes amino acids, an additional secretagogue, representing a more physiological test. Surrogate measures of insulin secretion and sensitivity based on glucose and insulin concentrations can be obtained during MMTTs and OGTTs (Table 1). Additionally, several disease risk indices can be estimated from the measurements of glucose, insulin and C-peptide during an OGTT.

**Table 1:** Measurable outcomes of dynamic and static metabolic tests in stage 1 and stage 2 type 1 diabetes

Test	Staging	FPIR	Insulin secretion	Insulin sensitivity	Beta cell function	Disease progression risk	Limits^a^
Dynamic tests							
Standard OGTT^b^	✓	✗	✗	✗	✗	✓	• $\uparrow$ Invasiveness • Age limit ($\ge$ 7–8 years)
Extended OGTT^c^	✓	✓	✓	✓	✓	✓	• $\uparrow$ Invasiveness • Age limit ($\ge$ 7–8 years)
MMTT	✗	✓	✓	✓	✓	✗	• $\uparrow$ Invasiveness • Age limit ($\ge$ 7–8 years)
IVGTT	✗	✓	✗	✗	✗	✓	• $\uparrow$ Invasiveness • Age limit ($\ge$ 7–8 years)
Hyperglycaemic clamp	✗	✓	✓	✓	✓	✗	• $\uparrow \uparrow \uparrow$ Invasiveness • Age limit ($\ge$ 7–8 years)
Euglycaemic–hyperinsulinaemic clamp	✗	✗	✗	✓	✗	✗	• $\uparrow \uparrow \uparrow$ Invasiveness • Age limit ($\ge$ 7–8 years)
Static tests							
Urinary C-peptide-to-creatinine ratio	✗	✗	?	✗	✗	?	• $\downarrow \downarrow \downarrow$ Invasiveness • No age limit
HbA_1c_/fasting glucose	✗	✗	✗	✗	✗	✓	• $\downarrow$ Invasiveness • No age limit
C-peptide capillary dried blood spot test	✗	✗	?	✗	✗	?	• $\downarrow$ Invasiveness • No age limit
Risk indices computed during OGTT							
DPTRS^d^	✗	✗	✗	✗	✗	✓	• $\uparrow$ Invasiveness • Age limit ($\ge$ 7–8 years)
Index60^e^	✗	✗	✓	✗	✗	✓	• $\uparrow$ Invasiveness • Age limit ($\ge$ 7–8 years)
Other							
CGM	✗	✗	✗	✗	✗	✓	• $\downarrow \downarrow \downarrow$ Invasiveness • No age limit

^a^$\downarrow$ relatively low, $\downarrow \downarrow \downarrow$ very low, $\uparrow$ relatively high, $\uparrow \uparrow \uparrow$ very high

^b^Standard OGTT: glucose is measured at 0, 1 and 2 h

^c^Extended OGTT: glucose, insulin and C-peptide are measured at multiple time points (seven or more) and the test can be prolonged up to 240 min. Early sampling (e.g. 10 and 15 min) allows FPIR to be estimated

^d^DPTRS= (1.57 × log BMI) − (0.06 × age [years]) + (0.81 × glucose summed from 30 to 120 min/100) − (0.85 × C-peptide summed from 30 to 120 min/10) + (0.48 × log C-peptide_0_)

^e^Index60 = 0.36953 × (log C-peptide_0_ [ng/ml]) + 0.0165 × glucose_60_ (mg/dl) - 0.3644 × C-peptide_60_ (ng/ml)

CGM, continuous glucose monitoring

## Integrated assessment of beta cell health and the use of modelling

The AUC C-peptide computed during an OGTT or MMTT has been largely adopted as a surrogate marker of beta cell function in most disease prevention trials targeting stage 1 [64] and stage 2 [9] type 1 diabetes, as well as in early-stage 3 type 1 diabetes [65–68]. While a relationship between AUC C-peptide and residual beta cell function has been described, the longitudinal trajectory of such a relationship is still debated [42, 69]. However, as discussed above, the lack of decline in AUC C-peptide until around 6 months before the clinical onset of disease [28] and the evidence for early impairment of insulin sensitivity [4–6] suggest that measures accounting for both C-peptide and glucose profile, as well as insulin action, may be more informative to track the disease trajectory and the efficacy of disease-modifier drugs in early-stage type 1 diabetes.

Supporting this hypothesis, an exploratory analysis conducted in unaffected family members of people with type 1 diabetes demonstrated that lower beta cell function (DI) characterised those progressing to later disease stages in the absence of measurable differences in AUC C-peptide [70]. Similarly, a post-hoc analysis conducted in participants in the TrialNet Abatacept study testing the effect of abatacept on disease progression in those with stage 1 type 1 diabetes [64] demonstrated that Index60—a composite measure of glucose and C-peptide—but not AUC C-peptide was able to show a favourable effect of the treatment after 12 months [71].

An original approach, accounting for both glucose and C-peptide response curves (GCRC) during an OGTT, was proposed in a recent analysis of the TNPTP that allowed the identification of GCRC ‘zones’ on the 2D grid plot in association with demographic, metabolic, autoantibody, HLA and risk data [45]. This approach suggested that a higher C-peptide level was a feature of participants with higher glucose levels, therefore pointing to a role of insulin resistance in disease progression.

## Modelling beta cell function during dynamic tests

Although C-peptide levels are a well-established surrogate measure for insulin secretion, it is worth noting that C-peptide metabolic clearance exhibits a certain interindividual variability under controlled experimental conditions. Furthermore, C-peptide levels must be interpreted with caution in renal failure, in which blood levels of C-peptide can be falsely elevated [72, 73] and, owing to its half-life (~30 min), may not reflect rapid fluctuations in insulin secretion shorter than 10 min that occur during a dynamic test such as an OGTT or MMTT. As such, metabolic models based on two or three ideal compartments that include C-peptide have been proven to better describe C-peptide kinetics and, in turn, the relationship between C-peptide plasma concentrations and actual insulin secretion. In a classical model, two-compartment kinetics assumes that C-peptide is distributed in a main compartment (plasma) and a peripheral compartment (extravascular space) in rapid equilibration. Two-compartment kinetics justifies the non-linear changes in peripheral C-peptide during the dynamic increase in insulin secretion measured in vivo [74, 75]. As a practical implication, a more accurate assessment of in vivo insulin secretion based on glucose and C-peptide measurements during a dynamic test such as an OGTT or MMTT is obtained when a two (or more) compartment model is applied, rather than using the raw data derived from the test [76, 77].

Metabolic models are simplified representations of actual physiology that allow the estimation of components of beta cell health based on a minimum dataset from a dynamic test [78–80]. Although several models of glucose, insulin and C-peptide kinetics have been proposed [12, 35, 81, 82], here we focus on Cobelli’s oral minimal model [83] and Mari’s model [82] because of their large validation in different age groups, including paediatric cohorts [5, 84], and wide use over the last few decades [35, 79, 81, 84, 85].

### The oral minimal model

This model provides a simplified description of the complex glucose and insulin physiology [86]. It adopts quasi-linear differential equations to estimate insulin secretion and sensitivity as a result of metabolic fluxes among different compartments. Briefly, one of the two equations in the model represents insulin kinetics in plasma and the other describes the effects of insulin and glucose itself on restoration of baseline glucose levels after its ingestion or intravenous administration. The model considers a ‘delay’ in insulin action on the target organs (liver and adipose tissue). The oral minimal model [83] is used to estimate insulin sensitivity (SI), beta cell responsiveness ($\varphi$_total_) and beta cell function (DI=SI x $\varphi$_total_). The three-compartment model has been previously validated against model-independent measurements using multiple tracer meal protocols and euglycaemic and hyperglycaemic clamps [84]. A reduced sampling protocol based on a 2 h OGTT and seven samples has been validated against the widely used 3 h nine-sample protocol, demonstrating accuracy in the estimates of $\varphi$_total_, SI and DI, thus paving the way to shorter and more suitable tests for screening procedures. The physiological underpinnings of the oral minimal model are outlined in Fig. 1 [24, 79]. Glucose-stimulated insulin secretion is made up of two components: a dynamic component, representing the secretion of readily releasable insulin, which is stimulated by the rate of increase in glucose concentration ($\varphi$_dynamic_), and a static component, which measures new insulin production in response to a given increment in glucose above basal concentrations ($\varphi$_*static*_) [77, 83, 86].

A major advantage of metabolic modelling is the possibility of estimating both insulin secretion and insulin sensitivity once serial measurements of glucose, C-peptide and insulin are obtained. The availability of early time points in dynamic testing (10 and 20 min or 15 min) is of pivotal importance in estimating early insulin release ($\varphi$_dynamic_) [18]. The major limitation of this metabolic modelling in larger populations is the need for qualified personnel to run the analysis and the requirement to obtain multiple samples during testing.

### Glucose sensitivity and the potentiation factor during oral dynamic tests

An alternative model-based strategy has been proposed by Mari and colleagues by introducing beta cell glucose sensitivity and the potentiation factor [81, 87]. Briefly, a first component of the insulin secretion model describes insulin secretion with respect to the glucose concentration during an OGTT/MMTT using a dose–response function. The mean slope of the dose–response curve over the measured glucose range is described as beta cell glucose sensitivity and is independent of insulin sensitivity. The dose–response curve is modulated by the so-called potentiation factor, which accounts for non-glucose stimuli, such as gut-derived incretin secretion, during the test. A second component of the insulin secretion model quantifies the dependence of insulin secretion on the rate of change in glucose concentration. This derivative component is described as the ‘rate sensitivity’ and is related to early insulin release [35, 81].

### Indices of risk for disease progression

Longitudinal OGTTs have long served to derive indices to stratify the risk for disease progression. Such an approach does not necessarily describe the underlying physiology of beta cell changes over time. Indices including dynamic changes in C-peptide or glucose are expected to perform better than those based on single time points or static measures. Combined risk scores including genetic, clinical and immunological characteristics generally outperform metabolic indices [88]; however, development and validation of such risk scores require large cohorts that can capture wider ranges of genetic risk and backgrounds.

Longitudinal studies have demonstrated that composite measures of both glucose and C-peptide are able to identify antibody-positive individuals with previously unrecognised metabolic abnormalities as being at risk of progressing to stage 3 type 1 diabetes. Examples of such composite measures are the Diabetes Prevention Trial–Type 1 Risk Score (DPTRS) and the Index60.

The DPTRS is a risk score derived by stepwise modelling based on univariate proportional hazards models, developed in islet cell autoantibody-positive individuals and validated in the TNPTP study. Designed to capture the increasing glucose concentrations within the normal range that occur years before diagnosis [53, 89] and the differing trends in the latter stages of progression in relation to post-challenge C-peptide and glucose levels, the DPTRS includes fasting C-peptide, summed OGTT C-peptide and glucose values from 30, 60, 90 and 120 min, and age and BMI [90, 91]. The change in DPTRS from baseline to 1 year was highly predictive of type 1 diabetes in participants in the DPT-1 trial [90], while a DPTRS value ≥7.00 was able to identify antibody-positive individuals within the normal glucose range at substantial risk for progression [92].

Index60, a solely metabolic index comprising the log fasting C-peptide, 60 min glucose and 60 min C-peptide values and derived similarly from univariate proportional hazards modelling within the DPT-1 and TrialNet Natural History Study (TNNHS) cohorts, has also demonstrated utility in identifying impending stage 3 type 1 diabetes among autoantibody-positive individuals with normal 2 h glucose values (<7.8 mmol/l) [93].

Risk indices remain a valuable tool for identifying those who will most likely progress to stage 3 type 1 diabetes; however, they do not describe the underlying metabolic changes, which can be measured through metabolic testing and modelling. Therefore, risk indices and metabolic measures can be seen as complementary, non-overlapping tools for the investigation of the early stages of type 1 diabetes [53, 54].

## Minimally invasive measures of beta cell health: continuous glucose monitoring

Continuous glucose monitoring (CGM) has shown promise in meeting the challenge of screening across different age groups owing to its minimal invasiveness, low cost and good acceptance level. There is growing evidence that CGM detects abnormalities in glucose control in children with stage 1 type 1 diabetes [94]. In a small study conducted in antibody-positive children [81], the presence of islet autoimmunity increased glycaemic variability and the percentage of time spent with blood glucose >7.8 mmol/l compared with antibody-negative children. In a larger TNPTP cohort, spending ≥5% of the time with blood glucose ≥7.8 mmol/l or ≥8.9 mmol/l resulted in a 2 year risk of progression to type 1 diabetes of 40% and 62%, respectively [95]. However, evidence from the TNPTP cohort has demonstrated that OGTT-derived metrics still have a higher discriminative ability to predict disease progression than CGM [96].

## Conclusion

Measures of C-peptide alone provide an incomplete portrait of beta cell function and disease progression during the early stages of type 1 diabetes, as they do not account for changing insulin sensitivity and the non-linear fluctuations in insulin secretion described in stage 1 and stage 2 type 1 diabetes.

While risk indices have proved to be a valuable tool for stratifying the risk of progression of disease, they provide limited quantitative evidence on actual functional beta cell mass and its longitudinal changes. On the other hand, deep metabolic phenotyping tests may require complex and burdensome procedures that may not be feasible across different age groups. Metabolic modelling of the data derived from standard tests such as the OGTT or MMTT provides a more accurate and convenient way to estimate both insulin secretion and insulin sensitivity in early-stage type 1 diabetes. Further validation of such models in larger longitudinal cohorts is needed to confirm the value of this approach for generating a rapidly responsive endpoint that could be used to accelerate therapeutic trials at this stage of the disease.

## Supplementary Information

Below is the link to the electronic supplementary material.

Supplementary file1 (PPTX 396 KB)

## Abbreviations

DI: Disposition index
DPT-1: Diabetes Prevention Trial – Type 1 Diabetes
DPTRS: Diabetes Prevention Trial–Type 1 Risk Score
FPIR: First-phase insulin response
GCPR: Glucose and C-peptide response curves
MMTT: Mixed meal tolerance test
TEDDY: The Environmental Determinants of Diabetes in the Young
TNPTP: TrialNet Pathway to Prevention
