# Correction: Quantifying beta cell function in the preclinical stages of type 1 diabetes

**DOI:** 10.1007/s00125-024-06335-w

**Published:** 2024-11-27

**Authors:** Alfonso Galderisi, Alice L. J. Carr, Mariangela Martino, Peter Taylor, Peter Senior, Colin Dayan

**Affiliations:** 1 Department of Pediatrics, Yale University, New Haven, CT USA; 2 Alberta Diabetes Institute, University of Alberta, Edmonton, AB Canada; 3 Division of Infection and Immunity, School of Medicine, Cardiff University, Cardiff, UK

**Correction: Diabetologia (2023) 66:2189-2199**

10.1007/s00125-023-06011-5

Unfortunately, the units for C-peptide in Fig. 2 were given as mmol/l instead of pmol/l. The corrected figure is reproduced below. The online version of the article has been corrected.

**Fig. 2:**
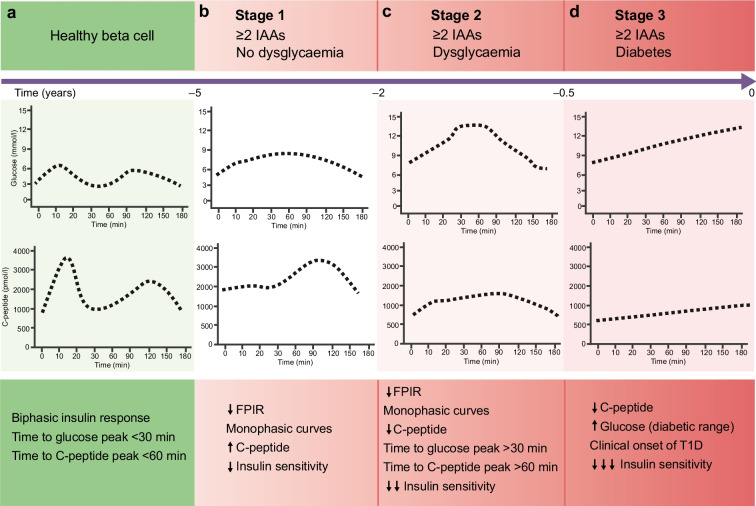
Glucose and C-peptide profiles during an OGTT through the progression from healthy beta cells (**a**) to stage 1 (**b**), stage 2 (**c**) and stage 3 (**d**) type 1 diabetes (T1D). The time (years) to diagnosis of type 1 diabetes is based on the available evidence and is intended as approximative. IAA, islet autoantibody. This figure is available as part of a downloadable slideset

