# Supplementary material for: Quantifying beta cell function in the preclinical stages of type 1 diabetes
Source: Diabetologia. 2023 Sep 15;66(12):2189–99. doi: 10.1007/s00125-023-06011-5 (PMC10627950; doi:10.1007/s00125-023-06011-5)
Supplement: Supplementary file 1 — Supplementary file1 (PPTX 396 KB) [file 125_2023_6011_MOESM1_ESM.pptx]

## Slide 1
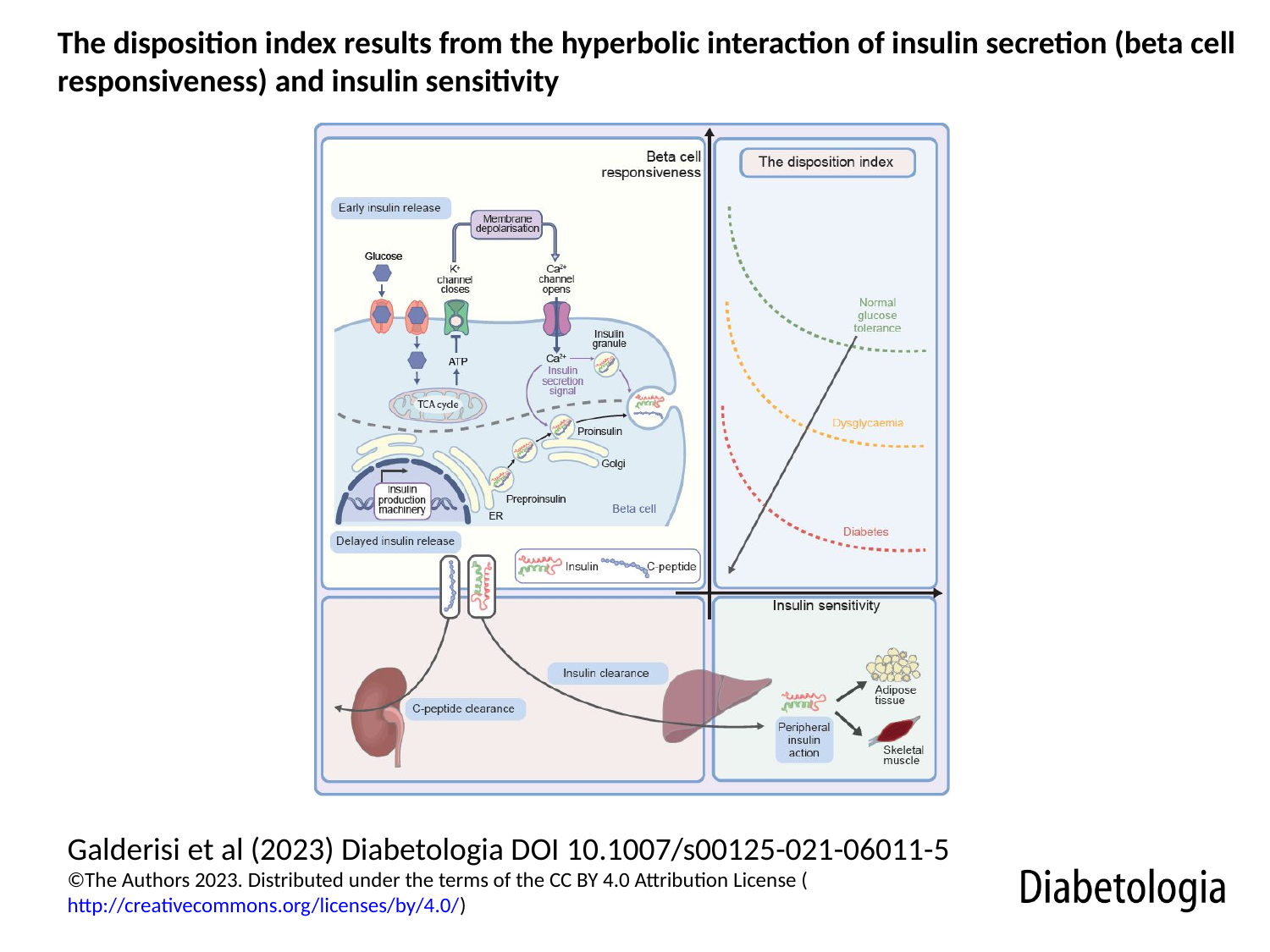

The disposition index results from the hyperbolic interaction of insulin secretion (beta cell responsiveness) and insulin sensitivity
Galderisi et al (2023) Diabetologia DOI 10.1007/s00125-021-06011-5
©The Authors 2023. Distributed under the terms of the CC BY 4.0 Attribution License (http://creativecommons.org/licenses/by/4.0/)

## Slide 2
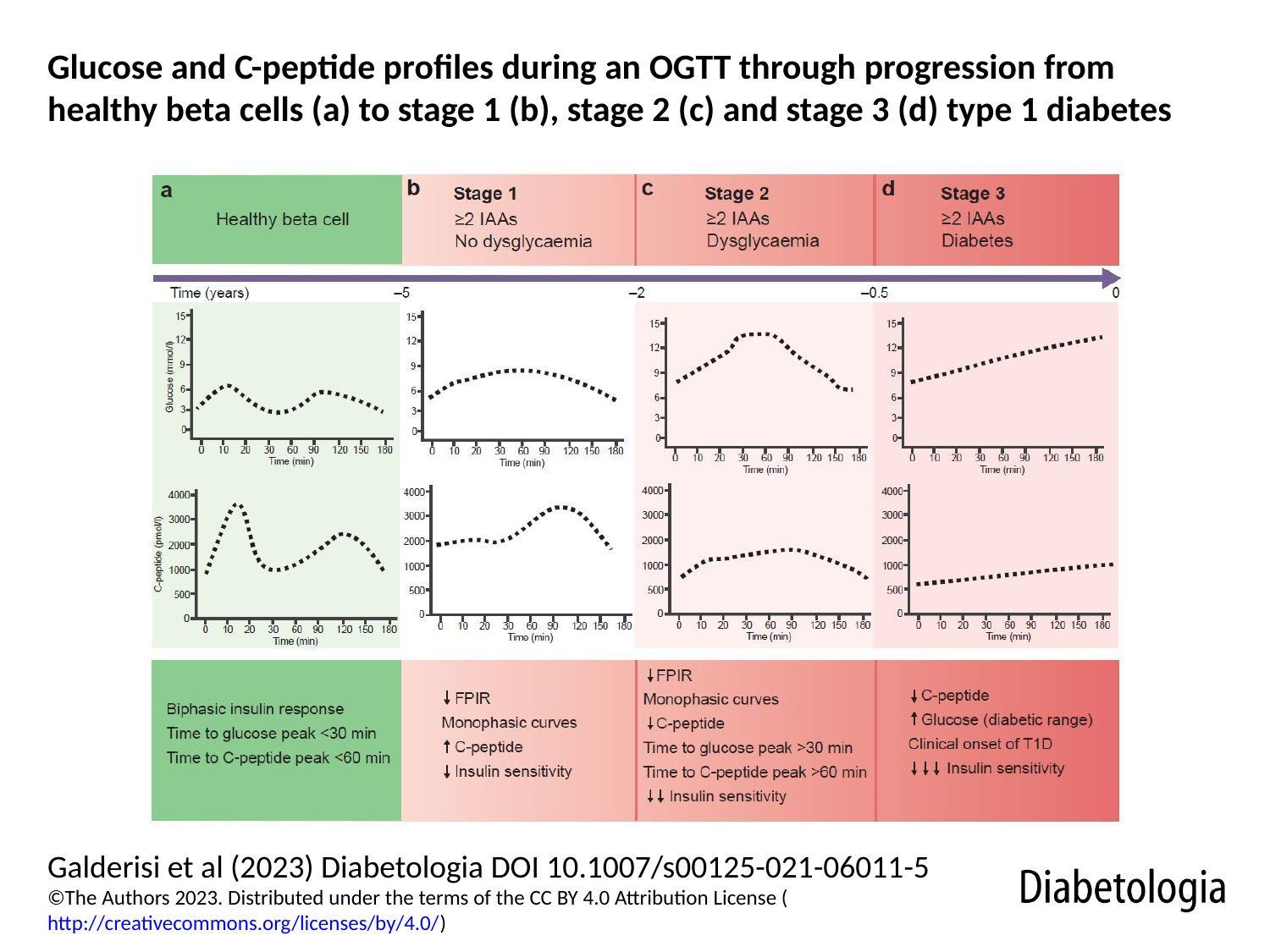

Glucose and C-peptide profiles during an OGTT through progression from healthy beta cells (a) to stage 1 (b), stage 2 (c) and stage 3 (d) type 1 diabetes
Galderisi et al (2023) Diabetologia DOI 10.1007/s00125-021-06011-5
©The Authors 2023. Distributed under the terms of the CC BY 4.0 Attribution License (http://creativecommons.org/licenses/by/4.0/)
